# Plasmapheresis for Suspected Drug-Induced Liver Injury During Pregnancy: A Multidisciplinary Diagnostic and Therapeutic Challenge

**DOI:** 10.3390/jcm14238385

**Published:** 2025-11-26

**Authors:** Agnieszka Zakrzewska, Magdalena Emilia Grzybowska, Dariusz Grzegorz Wydra, Natalia Katarzyna Mazur-Ejankowska, Krystian Adrych, Leszek Tylicki, Alicja Dębska-Ślizień, Bogdan Biedunkiewicz

**Affiliations:** 1 Department of Nephrology, Transplantology and Internal Medicine, Medical University of Gdansk, 80-210 Gdansk, Poland; agnieszka.zakrzewska@gumed.edu.pl (A.Z.); leszek.tylicki@gumed.edu.pl (L.T.); alicja.debska-slizien@gumed.edu.pl (A.D.-Ś.); bogdan.biedunkiewicz@gumed.edu.pl (B.B.); 2 University Clinical Centre, 80-952 Gdansk, Poland; magdalena.grzybowska@gumed.edu.pl (M.E.G.); dariusz.wydra@gumed.edu.pl (D.G.W.); krystian.adrych@gumed.edu.pl (K.A.); 3 Department of Gynecology, Obstetrics and Neonatology, Medical University of Gdańsk, 80-210 Gdansk, Poland; 4 First Doctoral School, Medical University of Gdansk, 80-210 Gdansk, Poland; 5 Department of Gastroenterology and Hepatology, Medical University of Gdansk, 80-210 Gdansk, Poland

**Keywords:** pregnancy, acute liver injury, drug-induced liver injury, plasmapheresis, hypertensive disorders of pregnancy, methyldopa

## Abstract

Acute liver injury during pregnancy is rare and predominantly associated with pregnancy-related conditions including acute fatty liver of pregnancy; Hemolysis, Elevated Liver Enzymes and Low Platelets syndrome; intrahepatic cholestasis of pregnancy; and preeclampsia. Drug-induced liver injury (DILI) is a less common and often overlooked cause of acute liver injury in pregnant patients. A literature search on acute liver injury during pregnancy and therapeutic plasma exchange was conducted, revealing the most common causative factors and syndromes. A 39-year-old woman was diagnosed with acute liver injury in the 23rd week of her third pregnancy and, upon extensive differential diagnoses, a suspicion of DILI occurred after the use of methyldopa. Methyldopa, the drug of choice for the treatment of hypertensive disorders of pregnancy, has a high safety profile for the developing fetus and is well tolerated by pregnant women, yet, in susceptible individuals, a hepatotoxic effect may occur. The drug was discontinued and symptomatic treatment with ursodeoxycholic acid and prednisone was implemented with marginal effect. Upon a multidisciplinary joint consultation, plasmapheresis procedures were introduced, granting a significant improvement in the patient’s liver function and enabling the continuation of the pregnancy. Plasmapheresis treatment was safely and effectively used for the first time in the therapeutic process in a pregnant patient with DILI. Interdisciplinary cooperation of specialists with gastroenterology, nephrology, and obstetrics expertise is crucial to achieving a timely diagnosis and begin effective treatment for pregnant patients with acute liver injury.

## 1. Introduction

Drug-induced liver injury (DILI) is currently the leading cause of acute liver failure in developed countries in the general population [1]. During pregnancy, about 2–3% of women with abnormal liver enzyme results are diagnosed with DILI [2]. Diagnosis of DILI is based on excluding other causes of liver damage: infectious, toxic, and genetic, as well as other gastrointestinal or autoimmune diseases. In the pregnant population, it is necessary to exclude liver-affecting diseases related to pregnancy, including acute fatty liver of pregnancy (AFLP) and Hemolysis, Elevated Liver Enzymes and Low Platelets (HELLP) syndrome, preeclampsia, and intrahepatic cholestasis of pregnancy (ICP).

The RUCAM (Roussel Uclaf Causality Assessment Method) scale, which is commonly used, helps to diagnose DILI, where points are assigned for clinical, biochemical, serological, and radiological features of liver injury, and the score reflects the likelihood that liver damage is caused by a specific drug [3]. Most of the cases of DILI reported in the literature regarding pregnancy focus on the idiosyncrasies of the drug used. Most often, adverse reactions are mild and not dependent on the dose of the drug or the duration of therapy [4]; fulminant liver failure requiring transplantation has been reported in isolated cases [5]. The mechanisms of DILI development are most often genetic polymorphisms responsible for high levels of the drug or its metabolite in blood or bile, decreased expression of protein transporters located in the tubular membrane, and/or decreased concentration of antioxidant compounds in hepatocytes, such as glutathione.

In individuals susceptible to liver damage caused by drug use, an indirect immune response occurs, which is an idiosyncratic reaction to protein–metabolite connections formed as a result of metabolic changes in the liver with the participation of cytochrome P450 [6]. This complex becomes a neo-antigen, and it is transported from the cell’s interior to the surface of the hepatocyte or cholangiocyte, where it triggers a cell-type response and penetrates into the bloodstream, stimulating a humoral response and inducing the production of antibodies directed against the modified drug–protein epitopes [7].

The basis of DILI treatment is discontinuation of the potential causative agent. In the case of cholestatic damage, ursodeoxycholic acid (UDCA) and/or cholestyramine are used to reduce bile acid levels by preventing their absorption, but the significance of this mechanism has not been proven [8]. The role of glucocorticoids (GCs) in the DILI treatment has not been proven and, according to most reports, the decision to initiate glucocorticoid therapy should be individualized [9]. In some cases, no therapeutic effect is observed despite the implemented pharmacological treatment, so additional therapeutic options should be explored.

Therapeutic plasmapheresis is a recognized treatment method for immunological and metabolic disorders and in cases of desensitization of immunized patients and in antibody-mediated rejection [10]. It consists of the extracorporeal separation of the morphotic elements of blood from the plasma to remove the plasma or cleanse it of pathological components. In the clinical setting, two treatment methods are used: therapeutic plasma exchange (TPE) and double-filtration plasmapheresis (DFPP). TPE is an extracorporeal procedure, during which the plasma is separated from the morphotic elements of the blood and then removed along with pathogens. It is estimated that to remove about 75% of harmful substances during one TPE treatment, about 2500–3500 milliliters of total plasma volume should be replaced [11]. DFPP, also known as selective plasmapheresis/cascade filtration, is the separation and removal of pathogenic components of a specific molecular weight from plasma. Colloids (freshly frozen plasma and/or albumin) or a combination of colloids and crystalloids are used as the replacement fluid in both methods. The common DFPP procedure is also called “one volume of plasma” replacement, which replaces approximately 63% of the patient’s initial plasma volume. The rationale for the use of TPE or DFPP in cholestatic liver injury is the removal of bile acids from plasma, which in turn allows for the removal of bile acids from peripheral tissues by equalizing the concentrations between the two compartments. The expected therapeutic effect after one DFPP session usually occurs within 2 days after the procedure and lasts up to 5 days [12].

## 2. Case Presentation

A prior-to-pregnancy healthy 39-year-old patient, in the 23^rd^ week of her third pregnancy, was admitted to the emergency department due to acutely onset pruritus of her hands and abdomen. Upon medical examination, a yellowish discoloration of the sclera and skin were observed, and the patient was admitted to the pathology of pregnancy ward on suspicion of jaundice of unclear etiology. Fetal ultrasonography (USG) revealed a normal-for-gestational age fetus with correct anatomy and correct fetal Doppler examination of umbilical artery, ductus venosus, and middle cerebral artery was confirmed.

Two months before the hospital admission, the patient was diagnosed with hypertension, and methyldopa at a dose of 250 milligrams was introduced daily with a good hypotensive effect. The patient had no prior jaundice or other liver diseases in her medical history. Her first pregnancy resulted in spontaneous pregnancy loss in the first trimester. Her second uncomplicated pregnancy took place 5 years before and resulted in a cesarean delivery of a healthy neonate. Her current pregnancy resulted from an in vitro fertilization.

Upon admission to the pathology of pregnancy ward, laboratory tests were alarmingly abnormal, including the following: bile acids of 432.4 µM/L; hyperbilirubinemia of 20.6 mg/dL; alanine aminotransferase (ALT) 2082 U/L; aspartate aminotransferase (AST) 3050 U/L; moderately elevated cholestatic enzymes: alkaline phosphatase (ALP) 166 U/L; gamma-glutamyl transferase (GGT) 67 U/L; hypoalbuminemia 22 G/L; prolonged International Normalized Ratio (INR) 4.95; and hyperammonemia 81 µmol/L. The results of laboratory tests are presented in Table 1.

Screening for infections was conducted, which included the following: hepatitis A, B, C, and E; cytomegalovirus (CMV); herpes simplex virus (HSV); Epstein–Barr virus (EBV), and human immunodeficiency virus (HIV); all results were negative of current infection.

An abdominal ultrasound was conducted, revealing a liver of a borderline size (craniocaudal distance of 14.7 cm, anterior–posterior distance of 13.5 cm), normal echogenicity, no focal lesions, and no dilated intrahepatic bile ducts were detected. Magnetic resonance imaging of the patient’s abdomen, performed in the following days, confirmed the ultrasound findings.

The consulting gastroenterologist recommended cessation of methyldopa, due to its potential hepatotoxic effect, which was previously described in the population of pregnant patients. UDCA treatment was initiated, initially at 16 mg/kg for 7 days, then gradually increased to 20 mg/kg for the next 16 days, up to a maximum of 30 mg/kg, which was used for the next 20 days, and then reduced to a maintenance dose of 10 mg/kg. It should be underlined that the standard dosage of oral UDCA for the treatment of ICP ranges from 10 to 15 mg/kg; however, due to the patient’s extremely high levels of bile acids, UDCA dosage was considerably increased.

Autoimmune hepatitis was suspected and, pending a panel of immunological tests, treatment with prednisone at a dose of 0.5 mg/kg bodyweight was initiated. A slight improvement in the patient’s clinical condition was achieved: a decrease in bile acid levels to 350 µmol/L, a decrease in bilirubin to 12.4 mg/dL, a decrease in transaminases ALT - 948 U/L, AST -1477 U/L, and a decrease of INR to 2.2.

To exclude liver diseases, numerous specialist tests were conducted, including the following: anti-smooth muscle antibodies (ASMAs) test for autoimmune hepatitis (AIH) and anti-bile duct antibodies test for primary biliary cirrhosis (PBC); all the results were negative. Only the determined titer of ANA-HEp-2 antinuclear antibodies showed an average positive titer of 1:640. Prednisone was continued at a maintenance dose of 0.25 mg/kg bodyweight. A liver biopsy was not performed due to a higher risk of bleeding during pregnancy, a high INR, and difficult access due to the enlargement of the uterus and a suspected DILI diagnosis.

On the seventh day of the hospitalization, due to the minimal clinical improvement of the patient’s condition, upon joint obstetrics, gastroenterology, and nephrology consultation, a decision to start treatment with plasmapheresis was made. Nine procedures were performed in total. The treatment tolerability was good, and no adverse events were observed. The first four treatments were performed everyday using the TPE method, in which the plasma exchange volume was 3 liters per treatment, and the average TBA reduction per single treatment was 104 µmol/L (33.32%). Another five treatments were performed every other day using the DFPP method. The planned filtration volume of a single treatment was also 3 liters, and the average TBA reduction per single treatment was 87.5 µmol/L (56.32%).

As a result of the treatment, a significant clinical improvement was achieved: a complete resolution of the pruritus and yellowing of the skin, and a biochemical improvement: the bile acid levels decreased to 5.4 µmol/L, bilirubin decreased to 2.95 mg/dL, and a normalization of transaminases and INR index was noted. ANA-HEp-2 titer decreased to 1:320. The effects of DILI treatment with plasmapheresis are presented in Table 1.

After the cessation of methyldopa, the patients’ blood pressure was monitored three times per day and no other hypotensive treatment was administered, as the blood pressure remained normal. It is key to underline that no hypotensive episodes were observed during TPE/DFPP treatment and cardiotocography was conducted three times per day and it remained appropriate for gestational age, without episodes of fetal tachycardia or bradycardia. Additional cardiotocography was conducted after each plasmapheresis session to monitor the fetal status, which remained stable and appropriate for gestational age. Short-term fetal heart rate variability was noted.

In the course of the hospitalization at the pathology of pregnancy ward, during the 27th week of gestation, due to the shortening of the cervix, the patient underwent a cervical cerclage. Later, during the 31st week of gestation, due to regular uterine contractions, tocolytic treatment of atosiban was introduced with a good therapeutic effect. The bile acid concentration at the time was 12 µmol/L. Upon the resolution of preterm labor symptoms and because of a stable liver status, the patient was discharged and monitored weekly at the pathology of pregnancy outpatient clinic.

The patient was readmitted to the clinic at 36 weeks of gestation due to preterm premature rupture of the membranes. An elective cesarean section was performed in delivering a healthy-in-neurological-examination boy, weighing 3290 grams, scoring 9/9/10 in Apgar Scale. Intraoperatively, the patient’s liver was visualized, and no abnormalities were noted.

Postnatal ultrasonography of the neonate’s brain and abdomen revealed no pathology. Blood tests performed by the neonatology specialist were normal, and after 9 days of observation, the mother and the newborn were discharged in good general condition.

Upon clinical reflection, it should be mentioned that the patient posed a diagnostic challenge, and the diagnosis and treatment would not be possible without a multidisciplinary cooperation and joint consultations, which led to the decision to initiate the plasmapheresis treatment. This was the first time that plasmapheresis was performed on a pregnant patient in our university hospital. The medical team faced some challenges regarding the transport of the patient from the Pathology of Pregnancy Department (where the patient was hospitalized due to monitoring the fetus by cardiotocography and ultrasonography) to the Nephrology Department where plasmapheresis took place.

## 3. TPE and DFPP Methods

The extracted plasma volume was calculated, using Nadler’s formula and hematocrit, in a range of 1.5 total plasma volume per session. DFPP was performed using the HF440 machine (Infomed SA, Geneva, Switzerland) with Granopen 060 Plasmafilter and Medopen 30 Plasmaseparator (Infomed SA, Geneva, Switzerland), with no necessity of replacement fluids. TPE was performed using the Multifiltrat machine (Fresenius, Bad Homburg, Germany) with plasmaFlux P2 dry Plasmafilter (Fresenius, Bad Homburg, Germany). As for volume replacement fluids, we used a 3000 mL 4% solution of human albumin. During the procedures, the patient received intravenous unfractionated heparin at a dose of 5000 U bolus and then 1000 U/h in the pump. There was no need to modify the treatment due to pregnancy.

## 4. Differential Diagnosis

Acute liver injury is highly uncommon during pregnancy and is predominantly caused by pregnancy related syndromes, including acute fatty liver of pregnancy (AFLP), Hemolysis, Elevated Liver Enzymes and Low Platelets (HELLP) syndrome, intrahepatic cholestasis of pregnancy (ICP), and preeclampsia. It is crucial to carefully consider pregnancy-related conditions in the differential diagnosis, as despite trials of pharmacological treatment, the only method of resolution is often through ending of the pregnancy and the delivery of the neonate. Cesarean section is often the chosen method of delivery, as induction of labor may be too lengthy and too high of a risk for the mother and the fetus. The characteristics of the diseases included in the differential diagnosis and our patient’s symptoms and results are presented in Table 2.

AFLP is a rare and often fatal condition, predominantly characterized by anorexia, nausea, jaundice, fever, and significant elevation of liver function tests leading to liver failure [13]. The pathogenesis of AFLP remains unclear, yet a fetal deficiency of long-chain 3-hydroxyacyl-coenzyme A dehydrogenase and changes in maternal hormone levels during pregnancy are considered among the risk factors. AFLP is diagnosed from the 28th to 40th week of gestation [13], and due to a later manifestation, AFLP was ruled out in the course of our differential diagnosis. Additionally, it is key to underline that patients diagnosed with AFLP meet the Swansea criteria, and hypertension is not a typical feature [13,14]. Interestingly, a cohort study of 298 patients describing treatment of AFLP with plasma exchange reported a reduction in maternal mortality [15], underling the safety of the procedure performed during pregnancy.

HELLP syndrome is a pregnancy-related condition usually diagnosed during the third trimester of pregnancy or shortly after the delivery. It has been considered a complication of preeclampsia (PE) [16], yet recent research questions this relationship and argues that HELLP may be separate from PE [17]. Two classifications are used in the diagnostic process of HELLP: Mississippi and Tennessee [18], both of which include the total platelets count, lactate dehydrogenase (LDH), as well as AST and ALT serum concentrations. It is necessary to underline that liver injury may be a feature of HELLP, predominantly through the formation of a sub-capsular liver hematoma, which may rupture in the course of the syndrome, increasing maternal and fetal mortality [19]. Crucially, patients suffering with HELLP syndrome may present with epigastric and/or upper-right quadrant pain, which is a symptom that should prompt for vigilance and a rapid diagnosis during pregnancy [16,19]. In accordance with American Society for Apheresis guidelines, TPE performed for postpartum and antepartum HELLP syndrome cases has the best results when it is initiated within 24 h of the diagnosis [20]. HELLP syndrome was ruled out in our patient due to a normal platelet count and no evidence of erythrocyte hemolysis as LDH was under 600 IU/I.

Intrahepatic cholestasis of pregnancy (ICP) is the most common liver disorder in pregnancy with a prevalence of 0.5–1.5% in Europe [21]. Different rates of ICP are observed depending on the geographical location and it is more common in South America and northern Europe [22]. The diagnostic criteria for ICP include pruritus with increased serum bile acid concentrations, and the diagnosis is common from late second trimester and early third trimester of pregnancy. Unfortunately, the etiopathology of ICP is not fully understood; however, precise monitoring of the condition is key to minimize the fetal complications through a timely administration of pharmaceutical treatment of UDCA. In severe cases of ICP, when pharmacotherapy is not sufficient, TPE has been used with success. ICP was high on the differential diagnosis chart for our patient. However, patients with ICP do not exhibit elevated bilirubin serum concentration exceeding 6 mg/dL [23]. TPE has been used in ICP therapy leading to the lowering of bile acids level and the improvement of pruritus; however, the use is restricted to women with the most severe manifestations [24].

Preeclampsia (PE) is a multi-organ pregnancy related condition, which results in the highest rates of maternal and fetal morbidity and mortality worldwide [25]. The etiology of PE remains widely researched, and the principal mechanism is utero-placental ischemia. The standard treatment of PE is mostly anti-hypertensive and anti-seizure management of magnesium sulfate therapy. A timely delivery is a key factor for maternal and fetal survival, and the treatment of PE should be conducted in reference centers with access to maternal–fetal medicine practitioners. PE is closely related to HELLP, and all patients diagnosed with PE should be regularly screened for HELLP and liver disfunction. TPE has, to date, been used in experimental treatment of severe early-onset PE with a good clinical response. The biggest study was conducted on a group of 20 pregnant women in a single-center study by Iannaccone et al. PE was ruled out in our differential diagnosis upon correct soluble fms-like tyrosine kinase-1/placental growth factor (sFlt-1/PIGF) ratio and the absence of proteinuria [26].

## 5. Discussion

Methyldopa is a centrally acting inhibitor of the α-adrenergic receptor. Currently, it is predominantly used in the treatment of hypertension during pregnancy, which is due to its established safety for the developing fetal–placental circulation. Chronic use of methyldopa is associated with mild and transient elevations of the serum transaminases in 5–35% of patients, which is most often resolved despite the continued use of the drug [27]. A clinically significant liver damage caused by methyldopa is relatively rare. The estimated risk of developing hepatotoxicity during the use of methyldopa in pregnancy is approximately 1%. [6]. The risk factors include age (young or advanced), gender (women are more likely to be affected), comorbidities (hepatitis, diabetes), nutritional status (malnutrition or obesity), gut microbiota composition, and genetic factors (CYP polymorphism, HLA variants) [28]. According to the Councils for International Organizations of Medical Sciences (CIOMS), DILI is classified, according to the R-value (R = (ALT/ALT × ULN)/(ALP/ALP × ULN), (ULN—upper limit of the norm), into three types: hepatocellular (R ≥ 5), cholestatic (2< R <5), and mixed (R ≤ 2). In most patients, the pattern of liver damage by methyldopa is hepatocellular, with marked 5- to 100-fold elevations in ALT, AST, and moderate increases in ALP. In a small percentage of patients, the pattern of damage is cholestatic or mixed [27]. Most often, the symptoms resemble those of acute viral hepatitis, including jaundice, headache, fatigue, anorexia, and nausea. Less commonly, fever, rash, joint pain, and eosinophilia may occur. Anti-erythrocyte auto-antibodies may be present in the serum, presenting a picture similar to autoimmune hemolytic anemia (AIHA) [29], antinuclear antibodies (ANAs), and anti-smooth muscle antibodies (SMA), and elevated levels of immunoglobulins (especially IgG) may be present [27]. After the discontinuation of the drug, most patients recover within 4 to 12 weeks [27].

For the case in question, acute liver injury manifested approximately 8 weeks after the start of α-methyldopa intake and was hepatocellular (R = 45 according to CIOMS). The patient scored 9 points on the RUCAM scale, which meant a high probability of DILI diagnosis (Table 3.). Due to the lack of improvement after the discontinuation of the drug, suspecting the immunological background of the observed disorders, prednisone therapy was implemented, obtaining only a slight improvement in the patient’s clinical condition. Due to a high serum bile acid concentration, their moderate reduction after UDCA use, and an increased risk of complications for the developing fetus, it was considered justified to initiate therapeutic plasmapheresis therapy.

It remains unknown whether the patient benefited more from the treatment with a non-standard, double-dose of UDCA and the rapid introduction of prednisone to treatment, from the plasmapheresis procedures, or the combined therapeutic strategies. Methyldopa was discontinued on the first day of the hospitalization, prednisone was initiated on day 4, and plasmapheresis commenced on day 6 and completed on day 21. Taking into consideration an idiosyncratic reaction to methyldopa (it either triggers a cell-type response or penetrates the bloodstream stimulating a humoral response with the production of antibodies directed against the modified drug–protein epitopes), the plasmapheresis treatment could be considered together with steroids in severe cases not responding to suspicious drug discontinuation and combined UDCA and steroids treatment.

## 6. Literature Review

**Figure 1:**
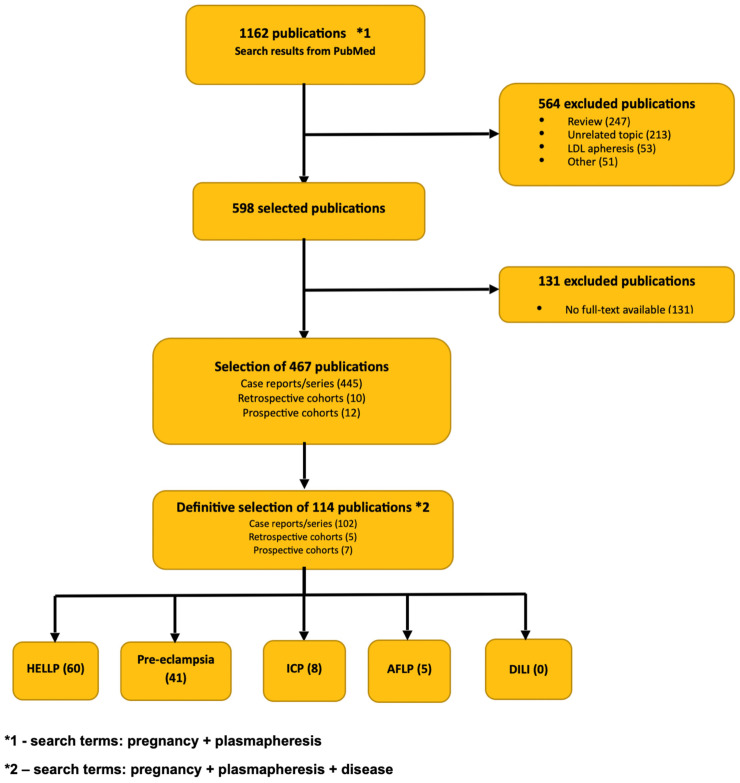
Flowchart of publications included in the literature review.

To identify clinical indications for the use of TPE in pregnant women with liver injury, we conducted a systematic literature search using a strategy that included the terms “plasmapheresis,” “double-filtration plasmapheresis,” and “plasma exchange” combined with the word “pregnancy,” followed by “liver injury” or “acute fatty liver of pregnancy” or “HELLP” or “preeclampsia” or “intrahepatic cholestasis of pregnancy.” The search included studies published until November 2025 in PubMed. Case reports (series), as well as retrospective and prospective studies in English language, were included. Double-filtration plasmapheresis and TPE were considered for the qualification in the literature review, excluding other plasma separation methods. To evaluate plasmapheresis treatment in pregnancy, data extraction from each study was performed separately by two independent investigators. A systematic literature review of plasmapheresis in the treatment of liver injury in pregnancy identified 116 publications (Figure 1). Of these, 60 were in patients treated for HELLP, 41 for preeclampsia, 8 for ICP, 5 for AFLP, 1 patient for Herpes Simplex Virus infection, and 1 patient for AIH. We found no publications regarding a pregnant woman with DILI treated with plasmapheresis.

The use of therapeutic plasmapheresis was explored in cases where standard treatment was insufficient for both pregnancy-related and unrelated diseases. The safety profile of TPE during pregnancy appears to be comparable to that of TPE in non-pregnant patients. Potential side effects are mainly due to a provoked drop in blood pressure, which could theoretically result in a reduced placental perfusion.

The level of evidence supporting the use of TPE in the general population with acute liver failure (ALF) is high. In 2016, the first multi-center, randomized control trial was published, which found that a 3-day TPE treatment improved the overall survival of ALF patients by 10% due to a weakening of the immune response, providing a homeostasis window for liver regeneration, as illustrated by improved systemic inflammatory response syndrome (SIRS) and sequential organ failure assessment (SOFA) scores [30].

The beneficial effect of TPE in DILI was evaluated retrospectively in a single-center study in 10 patients after different triggering medications. A total of 26 TPE procedures were performed with an average plasma exchange of 2.6 L per patient, achieving significant clinical improvement and significant reductions in bilirubin (*p* < 0.001), INR (*p* < 0.05), and transaminases (*p* < 0.05) [31]. Another retrospective study of 22 patients with DILI reported a 1-month graft-free survival of 59% (13/22 patients) using the low-volume plasma exchange (LVPE) in combination with a low-dose steroid. The one-month survival rates were 36%, 75%, and 86%, respectively, among DILI patients with hepatocellular, mixed, and cholestatic liver disease [32]. Currently, TPE is mainly used for thrombotic microangiopathies, lipid disorders, and various autoimmune diseases, the above-mentioned conditions in transplant candidates and recipients with antibody-mediated organ rejection [10].

A cohort study of 298 pregnant patients with AFLP was conducted [15]. Patients treated with TPE had a significantly lower 60-day mortality rate and a shorter duration of hospitalization than patients who were not treated with TPE. These findings are in line with a recently published systematic review and meta-analysis evaluating the effectiveness of plasmapheresis as an adjunctive treatment in patients with acute fatty liver of pregnancy (AFLP). A reduction in mortality and significant reductions in bilirubin, transaminases, and prothrombin time were reported with TPE [33]. Both techniques, TPE and DFPP, did not differ in terms of treatment tolerance, reduction in TBA concentration, and effect on the reduction in pruritus.

Patients with severe HELLP syndrome diagnosis benefit from early (within 24 h from the diagnosis) TPE and despite being a Category III and IV indication among postpartum and antenatal females [20]. A study by Chowdhry et al. [20] noted a significant increase in the platelet count, and a decrease in the lactate dehydrogenase levels (*p* < 0.05) was observed post TPE.

A study conducted by Ovadia et al. [24] explored TPE treatment for severe ICP, where the standard pharmacotherapy of UDCA was not effective in lowering the bile acid levels and decreasing the maternal symptom of pruritus. It was concluded that TPE can lower bile acids and improve the quality of life by the removal of pruritogens and a partial pruritus resolution. The authors highlighted that potential complications of TPE, along with the minimal sustained symptomatic benefit, restrict TPE use to the most severe IPC cases where other treatment options have been exhausted.

A study evaluating TPE therapy in an early-onset PE has been conducted in a group of 20 patients who underwent 95 TPE sessions [26]. TPE treatment enabled the continuation of pregnancy for 8.25 ± 5.97 days, compared to 3.14 ± 4.57 days in the control group (*p* = 0.004). The median sFlt-1/PlGF ratio was lowered by 18.02%. The fetal survival rate was higher in TPE-treated cases than in the control group who received the standard treatment, and Neonatal Intensive Center Unit hospitalization was shortened by the median of 15 days. It should be highlighted that the monocentric study reports the largest experience with TPE treatment of PE worldwide [26]. Treatment of PE with TPE is not a standard clinical practice and multi-center studies are required to further investigate its effects and potential complications.

## 7. Conclusions

TPE was safely and effectively used for the first time in the treatment of DILI during pregnancy. It should be underlined that we present a single, uncontrolled case report with overlapping therapies of DILI including TPE, UCDA, and steroid therapy. The intensive treatment was crucial for the continuation of the pregnancy and to reduce the risks of fetal prematurity. Further studies and evidence are needed to clarify the indications for the use of TPE in severe drug-induced liver injury in pregnant women.

Pregnant patients presenting with jaundice require immediate hospital admission, differential diagnosis, and treatment. Pregnancy-related causes of acute liver injury including AFLP, HELLP, ICP, and preeclampsia should be considered along with other factors of infectious, toxic, drug-induced, genetic, and autoimmune etiologies. A pregnancy complicated by maternal acute liver injury poses a substantial threat to fetal development and requires intensive treatment and obstetrical monitoring to avoid maternal and fetal complications.

## Figures and Tables

**Table 1:** The results of laboratory tests correlated with medical interventions during the treatment—a timeline of hospitalization.

Day of Treatment	1.	2.	3.	4.	5.	6.	7.	8.	9.	10.	12.	16.	18.	**20.**	**22.**	**30.**	**50.**
Medical Intervention	Admission Methyldopa discontinuation UDCA initiation	UDCA	UDCA	UDCA Prednisone Initiation	UDCA Prednisone	UDCA Prednisone	UDCA Prednisone First TPE	UDCA Prednisone Second TPE	UDCA Prednisone Third TPE	UDCA Prednisone Fourth TPE	UDCA Prednisone First DFPP	UDCA Prednisone Second DFPP	UDCA Prednisone Third DFPP	UDCA Prednisone Fourth DFPP	UDCA Prednisone Fifth DFPP	UDCA	UDCA
TBA [µmol/L] Normal value <6	224	-	350	432	412	350	324	251 212	231	157	263 103	276	160 158	131 22	62 31	10	5
BIL [mg/dL] Normal value 0.1–1.2	17.3	-	20.5	-	14.6	12.4	7.6	-	-	-	14.7	-	-	-	-	4.5	2
ALB [g/L] Normal value 35–50	22	-	-	-			-	29	34	35	28	-	33	-	-	-	-
AST [U/L] Normal Value 11–34	3050	2757	3059	2764	-	948	825	661	-	-	241	-	-	116	61	52	39
ALT [U/L] Normal Value <34	2086	1892	1949	-	1824	1477	-	463	-	-	149	-	-	103	47	48	36
GGT [U/L] Normal value <38	57	52	-	-	-	53	-	-	-	-	31	-	-	-	-	-	36
ALP [U/L] Normal value 46–122	166	152	-	-	119	-	-	72	-	-	-	-	-	-	-	-	-
INR Normal Value 0.9–1.3	1.33	-	1.85	4.95	-	-	2.20	-	2.40	-	1.21	1.15	1.05	1.26	0.92	0.99	-
LDH [U/L] Normal Value 125–220	563	454	557	424	-	246	215	-	190	-	216	144	-	141	-	48	13
Ammonia [µmol/L] Normal Value 18–72	81	-	79	81	-	-	-	-	-	-	-	-	-	-	-	58	-

**Table 2:** Presentation of diseases included in the differential diagnosis and our patient’s symptoms and results.

Disorder of Pregnancy	Time of Onset	Risk Factors	Symptoms	Liver Enzimes	Platelet Count	Bile Acids	Hypertension/Proteinuria
AFLP	Typically from 28th to 40th week of pregnancy	Multifetal pregnancy, male fetus, co-existing liver disorders, history of AFLP, diabetes mellitus	Jaundice, nausea, vomiting, abdominal pain, polydipsia/poliuria, fatigue, fever, anorexia, ascites	ALT and ASPAT from 1 to 3× norm, up to 200 U/L	Thrombocythopenia +/−	Normal	20–40% of patients
HELLP	Typically from 27th week of pregnancy and in early postpartum	Nulliparity, multifetal pregnancy, maternal age > 40 years old, BMI > 35, previous history of HELLP	Abdominal pain, headache, nausea, vomiting, fatigue	ALT and ASPAT from 1 to 100× norm	Thrombocytopenia present usually from 50 to 150 G/L	Normal	85% of patients
ICP	Typically in the 3rd trimester of pregnancy, rarely before 26th week of gestation	History of liver disease, multifetal pregnancy, advanced maternal age, in vitro fertilization	Pruritus, darker urine, jaundice in less than 10% of women	ALT and ASPAT from 1 to 100× norm	Normal	Increased	No
Preeclampsia	Typically after 24th week of gestation	History of preeclampsia, hypertension, diabetes, kidney disease, autoimmune conditions, multifetal pregnancy, first pregnancy, BMI > 35, in vitro fertilization	Abdominal pain, vomiting, headaches, peripheral edema, visual disturbances	ALT and ASPAT from 1 to 5× norm	Thrombocytopenia present	Normal	Yes
DILI	Typically after 1st trimester of pregnancy	Advanced age, obesity, history of liver disease, female sex, diabetes, alcohol, smoking, pregnancy	Jaundice, abdominal pain, pruritus, fatigue, nausea, vomiting	ALT and ASPAT usually over 2× norm	Possible thrombocytopenia	Increased	No
Our Patient	23rd week of pregnancy	39 years old, in vitro fertilization, gestational hypertension, BMI 33, singleton pregnancy, no history of liver disease	Jaundice, pruritus	ALT 2083 U/L; ASPAT 3050 U/L	Normal, >150 G/L	Extremely high, 251 umol/L	Yes

**Table 3:** Presentation of patient’s score in the RUCAM scale assessing probability of DILI diagnosis.

RUCAM Scale: DILI Hepatocellular Type	Score
Time to onset from the beginning of drug intake: 5–90 days	2
After stopping the drug, change in ALT between peak value: decrease ≥ 50% within 8 days	3
Exclusion of other causes of liver injury: Acute viral hepatitis due to HAV (IgM anti-HAV); HBV (HBsAg and/or IgM anti-HBc); HCV (anti-HCV and/or HCV-RNA with appropriate clinical history); Biliary obstruction (by imaging); Alcoholism (history of excessive intake and AST/ALT ≥ 2); Recent history of hypotension, shock, or ischemia (within 2 weeks of onset); Complications of underlying disease(s) such as autoimmune hepatitis, sepsis, chronic hepatitis B or C, primary biliary cirrhosis, or sclerosing cholangitis; Clinical features or serologic and virologic tests indicating acute CMV, EBV, or HSV.	2
Previous information on hepatotoxicity of the drug: reaction labeled in the product characteristics	2
Total score	9

## Abbreviations

AFLP	Acute Fatty Liver of Pregnancy
AIHA	Autoimmune Hemolytic Anemia
ALF	Acute Liver Failure
ALP	Alkaline Phosphatase
ALT	Alanine Aminotransferase
ANA	Antinuclear Antibody
ASMA	Anti-Smooth Muscle Antibody
AST	Aspartate Aminotransferase
CIOMS	Council for International Organizations of Medical Sciences
CMV	Cytomegalovirus
DILI	Drug-Induced Liver Injury
DFPP	Double-Filtration Plasmapheresis
EBV	Epstein–Barr Virus
GCs	Glucocorticoids
GGT	Gamma-Glutamyl Transferase
HBV	Hepatitis B Virus
HCV	Hepatitis C Virus
HEp-2	Human Epithelial Type 2
HIV	Human Immunodeficiency Virus
HSV	Herpes Simplex Virus
ICP	Intrahepatic Cholestasis of Pregnancy
IgG	Immunoglobulin G
INR	International Normalized Ratio
LVPE	Low-Volume Plasma Exchange
PBC	Primary Biliary Cirrhosis
PE	Preeclampsia
RUCAM	Roussel Uclaf Causality Assessment Method
sFlt-1/PlGF	Soluble Fms-like Tyrosine Kinase-1 / Placental Growth Factor
SMA	Smooth Muscle Antibody
SIRS	Systemic Inflammatory Response Syndrome
SOFA	Sequential Organ Failure Assessment
TBA	Total Bile Acid
TPE	Therapeutic Plasma Exchange
UDCA	Ursodeoxycholic Acid
ULN	Upper Limit of Normal
